# Early Bleeding After Laparoscopic Roux-en-Y Gastric Bypass: Incidence, Risk Factors, and Management — a 21-Year Experience

**DOI:** 10.1007/s11695-022-06173-y

**Published:** 2022-08-06

**Authors:** Maja Odovic, Daniel Clerc, Nicolas Demartines, Michel Suter

**Affiliations:** 1 Department of Visceral Surgery, Lausanne University Hospital (CHUV), Rue du Bugnon 46, 1011 Lausanne, Switzerland; 2 Faculty of Biology and Medicine, University of Lausanne, Rue du Bugnon 21, 1005 Lausanne, Switzerland; 3 Department of Surgery, Riviera-Chablais Hospital, Route du Vieux Séquoia 20, 1847 Rennaz, Switzerland

**Keywords:** Roux-en-Y gastric bypass, Obesity, Laparoscopy, Bleeding, Complications

## Abstract

**Purpose:**

Morbidity and mortality associated with bariatric surgery are considered low. The aim of this study is to assess the incidence, clinical presentation, risk factors, and management of early postoperative bleeding (POB) after laparoscopic Roux-en-Y gastric by-pass (RYGB).

**Materials and Methods:**

Retrospective analysis of prospectively collected data of consecutive patients who underwent RYGB in 2 expert bariatric centers between January 1999 and April 2020, with a common bariatric surgeon.

**Results:**

A total of 2639 patients underwent RYGB and were included in the study. POB occurred in 72 patients (2.7%). Intraluminal bleeding (ILB) was present in 52 (72%) patients and extra-luminal bleeding (ELB) in 20 (28%) patients. POB took place within the first 3 postoperative days in 79% of patients. The most frequent symptom was tachycardia (63%). Abdominal pain was more regularly seen with ILB, compared to ELB (50% vs. 20%, respectively, *p* = 0.02). Male sex was an independent risk factor of POB on multivariate analysis (*p* < 0.01). LOS was significantly longer in patients who developed POB (8.3 vs. 3.8 days, *p* < 0.01). Management was conservative for most cases (68%). Eighteen patients with ILB (35%) and 5 patients with ELB (25%) required reoperation. One patient died from multiorgan failure after staple-line dehiscence of the excluded stomach (mortality 0.04%).

**Conclusion:**

The incidence of POB is low, yet it is the most frequent postoperative complication after RYGB. Most POB can be managed conservatively while surgical treatment is required for patients with hemodynamic instability or signs of intestinal obstruction due to an intraluminal clot.

**Graphical abstract:**

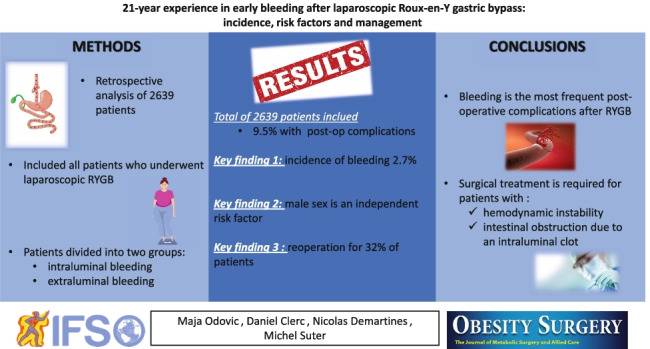

## Introduction

The overall prevalence of obesity worldwide is estimated to be 13% (https://www.who.int/news-room/fact-sheets/detail/obesity-and-overweight), and around 256,000 bariatric surgical procedures were performed in the USA in 2019 (https://asmbs.org/resources/estimate-of-bariatric-surgery-numbers). The most commonly performed procedure is vertical sleeve gastrectomy (SG) (59%) followed by laparoscopic Roux-en-Y gastric bypass (RYGB) (18%). In Switzerland, the situation is reversed, with RYGB being the most frequently performed procedure (75–80%), followed by SG.

The most frequent complications after RYGB are post-operative bleeding (POB), anastomotic leak, thrombo-embolic events, respiratory complications, infections, and intestinal obstruction. POB is reported with a rate of 0.9–4.4%, with a clear tendency to a lower rate in recent studies [1–5]. Bleeding can be of various origins: intra-luminal bleeding (ILB) related to bleeding from the staple-lines or an acute marginal ulcer at the gastrojejunostomy (GJ), whereas extra-luminal bleeding (ELB) refers to bleeding occurring in the abdominal cavity or from the port-sites. Several technical recommendations have been described to minimize POB incidence: the use of 3.5-mm circular stapler height for confection of the GJ [6–8], bidirectional Tri-Staple technique for the confection of the jejunojejunostomy (JJ) [9] and oversewing of the staple lines [10].

The aim of the present study was to access the incidence, clinical presentation, type of early POB following RYGB, identify risk factors, and describe management strategies.

## Material and Methods

### Patients

Our prospectively maintained bariatric surgery database was reviewed for all patients who underwent RYGB gastric bypass between 1999 (first case) and April 2020 in two reference bariatric centers with a common bariatric surgeon. Patients operated with a primary open approach were excluded. Standardized preoperative preparation was applied for all patients, before indication for surgery was confirmed [8]. Patients who presented early postoperative bleeding (POB), defined as occurring within 30 days of the index procedure, were included in the study, and compared to patients without bleeding. All patients are included in the COOL (COhorte Obésité Lausannoise) [11] cohort and gave informed consent for deidentified use of their data. Using data from the COOL cohort was approved by the local ethics committee (decision CER-VD 304/15).

### Surgical Technique

All procedures were performed in the lithotomy position using a 6-port technique, a 45° angled scope, and standard laparoscopic instruments. For all patients, surgery was performed by, or, under the direct supervision of one of 2 fully trained bariatric surgeons. The surgical technique has been previously published in details [8]. In our early experience, gastric transection was performed using a linear stapler with two-row 3.5-mm height staples. Since 2010, three-row endoscopic staplers (Tri-Staple® staplers, Covidien, Dublin, Ireland) were introduced, combining three different staplers’ heights (inner to outer row: 3 mm, 3.5 mm, 4 mm). Except in our first 70 patients, the excluded stomach staple line was routinely oversewn with a running absorbable suture. The GJ was performed using a 21-mm circular stapler with the anvil inserted transorally, using two-row 3.5-mm height staples. A side-to-side JJ was performed using a linear stapler with 2.5-mm height staples (two-row until 2010, then three-row thereafter) and the enterotomy closed with an absorbable running suture.

### Perioperative Care

Thromboembolic prophylaxis was initiated using low-molecular weight heparin administered 12 h before surgery, or using unfractioned heparin during induction of anesthesia, and then pursued for 2 weeks using low-molecular weight heparin. The preoperative doses were as follows: 40 mg/d enoxaparin for patients with a body weight < 150 kg, 60 mg between 150 and 200 kg, and 80 mg above 200 kg. In case unfractioned heparin was used before incision, 5000 units were administered with body weight < 150 kg, and 7500 units above this limit. Additional prophylaxis was provided using gradual compression stockings (replaced by intermittent pneumatic compression devices in 2010) during surgery and until full mobilization. In patients with previous thromboembolism history, or with a known hypercoagulable condition, the dose of enoxaparin was increased of 20 mg per day and pursued 4 weeks after surgery. For patients under chronic anticoagulant therapy, therapy was stopped a few days before surgery and switched to low-molecular weight heparin, with prophylactic dosing the day before surgery [12]. For patients under anti-aggregating agent, therapy was maintained during the perioperative period except on the day of surgery and postoperative day 1.

### Outcomes

All postoperative complications developing during the first 30 days following surgery were recorded and classified [13]. Patients with POB were either identified clinically and/or by a drop of the hemoglobin level. Patients underwent diagnostic tests according to their clinical condition using clinical examination, CT scan, upper gastrointestinal endoscopy, laparoscopy, laparotomy, or a combination thereof.

POB was divided in 2 groups: intraluminal bleeding (ILB) and extraluminal bleeding (ELB).

### Statistical Analysis

All statistical analysis were done using the STATA v16.1 software for windows (Statacorp, College Station, Tx, USA). Chi-square and Student’s *t*-test were used to compare categorical and continuous variables, respectively. Factors identified as predictors after univariate analysis were introduced in a multivariate logistic regression model. Statistical significance was accepted with *p* < 0.05.

## Results

During the study period, 2639 patients underwent laparoscopic RYGB in our two institutions, of whom 253 (9.5%) presented at least one postoperative complication. The most frequent complications were POB (2.7%), leak (1.1%), and wound infection (1.1%) followed by intestinal obstruction without hemorrhage (0.8%), intra-abdominal infection (0.8%), and respiratory infection and/or pleural effusion (0.8%). Of 72 (2.7%) patients with POB, 52 patients presented ILB (72%) and 20 presented ELB (28%). Patients’ characteristics are described in Table 1. On univariate analysis, compared to patients without POB, POB was associated with male sex (37.5% vs 23.6%, *p* = 0.01), older age (44.9 vs 42.1 years, *p* = 0.04), and pre-existing high blood pressure (59.7% vs 46.9%, *p* = 0.04). After multivariate analysis, only male sex (*p* = 0.01) remained an independent risk factor. Among patients with POB, 3 (4%) were under chronic anticoagulant therapy and 9 (13%) were on a single anti-aggregating agent, which was maintained among all but one. One patient had clopidogrel 75 mg/d, stopped one week prior to surgery. In all patients, routine coagulation tests (prothrombin time, partial thromboplastin time) as well as platelet count were normal before surgery. LOS was significantly longer in patients who developed POB (8.3 vs 3.8 days, *p* < 0.01).

**Table 1:** Patients’ characteristics

	Overall	No POB	POB	*p*-value
Patients	2639	2567 (97.3%)	72 (2.7%)	-
Male	634 (24%)	607 (23.6%)	27 (37.5%)	**0.01**
BMI (kg/m^2^)	44.4 (6.3)	44.5 (6.3)	43.7 (7)	0.39
*- Superobesity*	459 (17.4)	449 (17.5)	10 (14.1)	0.52
Age (years)	42.2 (11.4)	42.1 (11.3)	44.9 (12.4)	**0.04**
Comorbidities				
*- Hypertension*	1225 (47.1)	1182 (46.9)	43 (59.7)	**0.04**
*- Sleep apnea syndrome*	1453 (56)	1437 (56)	39 (54.2)	0.81
- Abnormal glucose metabolism**	1762 (66.7)	1715 (68.4)	47 (65.3)	0.60
*- Hypercholesterolemia*	1778 (67.4)	1734 (69.6)	44 (64.7)	0.42
*- Hypertriglyceridemia*	884 (34.8)	863 (34.9)	21 (31.3)	0.60
Duration of surgery (minutes)	148 (44)	148 (44)	144 (41)	0.52
LOS (days)	3.9 (3.7)	3.8 (3.3)	8.3 (10.3)	** < 0.01**
RYGB as a reoperation	253 (9.6)	146 (9.6)	5 (6.9)	0.54

Data is expressed as n (%) or mean (SD). *BMI* body mass index, *LOS* length of hospital stay, *POB* post-operative bleeding, *RYGB* laparoscopic Roux-en-Y gastric by-pass. *Superobesity defined as *BMI* $\ge$ 50 kg/m.^2^. ** includes patients with diabetes, prediabetes and insulin resistance

Most patients presented POB within the first 3 postoperative days (79.2%) and during index hospitalization, whereas 7 patients (10%) needed readmission for POB. The most frequent symptom was tachycardia in 45 (63%) patients. Abdominal pain was more frequent with ILB compared with ELB (50% vs. 20%, *p* = 0.02). A median drop in hemoglobin level of 46 g/L (1–88) was found, without difference between ILB and ELB (*p* = 0.85) (Table 2).

**Table 2:** Clinical presentation of bleeding

	All patients with POB (= 72)	ILB (*n* = 52)	ELB (*n* = 20)	*p-*value
Bleeding onset (days)	1 (0–23)	2 (0–23)	1 (0–7)	0.12
Coagulation status				
*- Antiaggregating therapy*	11 (15%)	7 (13%)	4 (20%)	0.49
*- Anticoagulation therapy*	3 (4%)	2 (4%)	1 (5%)	0.83
Clinical presentation				
*- Tachycardia*	45 (63%)	32 (62%)	13 (65%)	0.79
*- Hematochezia*	32 (46%)	32 (63%)	0	*-*
*- Abdominal pain*	30 (42%)	26 (50%)	4 (20%)	**0.02**
*- Hypotension*	23 (33%)	14 (27%)	9 (47%)	0.14
*- Hematemesis*	18 (25%)	18 (35%)	0	*-*
Hemoglobin (g/L)				
*- Preoperative*	141 (108–176)	140 (108–175)	140 (130–176)	0.26
*- Postoperative*	93 (55–151)	93 (55–146)	95 (60–151)	0.53
*- Hemoglobin drop*	46 (1–88)	46 (6–86)	41 (1–88)	0.85

Data is expressed as n (%) or median (range). *POB* post-operative bleeding, *ILB* intra-luminal bleeding, *ELB* extra-luminal bleeding

Investigations included upper gastrointestinal endoscopy in 8 patients, mostly with hematemesis. This showed a marginal ulcer in 3, a Mallory-Weiss tear in one, bleeding from the GJ staple in 2, and was negative in 2. CT scan was performed in 41 patients. Findings were free intra-abdominal fluid in 9 patients, suspicion of an intraluminal clot in 8, with probable active bleeding in 1, dilatation of the excluded stomach in 12, a large hematoma in the abdominal wall in one. No abnormality was seen in the remaining 11 patients.

Initial management for both groups included withdrawal of anticoagulant medication, hemodynamic support, pro-coagulants (Etamsylate, 500 mg 4 × /day), and blood transfusion as required. Transfusion was required for 41 (57%) patients. Median number of RBC packs was 2 (range 0–18). Reoperation was necessary for 23 (32%) patients. Two reoperations required conversion to open surgery, and the other 21 were completed laparoscopically (Table 3).

**Table 3:** Bleeding management

	All patients with POB (*n* = 72)	ILB (*n* = 52)	ELB (*n* = 20)	*p*-value
Anticoagulation withdrawal	61 (86%)	47 (90%)	14 (74%)	**0.03**
*-Duration (days)*	2 (0–7)	2 (0–7)	3 (0–6)	0.86
Etamsylate treatment	32 (44%)	24 (46%)	8 (40%)	0.64
Transfusion	41 (57%)	26 (50%)	15 (75%)	0.05
*-RBC units*	2 (0–18)	1 (0–18)	2 (0–7)	0.46
Reoperation	23 (32%)	18 (35%)	5 (25%)	0.43
*-Time to reoperation (days)*	1 (0–26)	1 (1–26)	6.5 (0–7)	0.99

Data is expressed as n (%) or median (range). *POB* post-operative bleeding, *ILB* intra-luminal bleeding, *ELB* extra-luminal bleeding, *RBC* red blood cells

One female patient with ILB and obstruction due to intraluminal clotting within the excluded stomach developed a leak from the remnant staple line with severe peritonitis and septic shock. She eventually died of irreversible brain damage due to a prolonged circulatory arrest during induction of anesthesia before reoperation. She represents the only postoperative mortality in our entire series.

### Management of Intraluminal Bleeding

The source of bleeding was identified with certainty in 22 patients. It originated from the GJ in 6 patients, the excluded stomach in 4 patients, and from the JJ in the remaining 12. Two patients with bleeding in the gastric remnant also developed a leak from the latter, and one patient with hemorrhage at the gastro-jejunostomy developed a leak at this level. Eleven patients developed an obstruction at the level of the JJ due to the accumulation of blood clots, with dilatation of the biliopancreatic limb and gastric remnant.

Eighteen patients with ILB required urgent reoperation either because of hemodynamic instability or intestinal obstruction. A laparotomy was done in the patient with septic shock because a laparoscopic approach was deemed too dangerous, and in another patient to allow for intragastric exploration, which proved negative. In this patient who developed recurrent bleeding, angiography eventually showed bleeding from an arteriovenous malformation in the stomach wall, which was successfully treated by embolization. Reoperation was completed by laparoscopy in the remaining patients. In 14 of these patients, a tube gastrostomy was done initially to empty the stomach before intraluminal blood clots were evacuated via an enterotomy just distally to the jejuno-jejunostomy. The gastrostomy tube was closed as soon as normal transit through the jejuno-jejunostomy resumed and was removed in the outpatient clinic after 4-\–5 weeks. In one patient with bleeding in the remnant but without distension, the distal gastric suture line was simply oversewn. In the last patient, the JJ was reopened to achieve hemostasis with cautery and the anastomosis was redone. Except the aforementioned patient who died, all patients fully recovered with no long-term sequela.

### Management of Extraluminal Bleeding

Twenty patients developed ELB of whom 5 required reoperations. Two were reoperated on the day of surgery because of hemodynamic instability and hemostasis was achieved using cautery on a short splenic vessel in one, at a trocar site in the other. Three patients required re-laparoscopy because of progressive abdominal pain, with positive cultures of the abdominal fluid in two. In the remaining patients who were stable with minor symptoms, bleeding stopped either spontaneously or after temporary withdrawal of prophylactic anticoagulation or after introduction of etamsylate. All patients with ELB recovered completely.

## Discussion

In the present series, early postoperative bleeding occurred in 2.7% of patients. This rate is consistent with the reported incidence in the literature of 0.9–4.4% [1–5, 14–17]. We found an incidence of bleeding lower in the most recent studies and we consider these results are due to enhancement of the surgical technique together with improvement of surgical instruments (staplers and energy devices). Tachycardia was the most prevalent sign or symptom of the bleeding events, occurring in 63% of patients in our study, similar with other authors reporting presence of tachycardia in up to 60% [1, 18]. The identified risk factors after univariate analysis were male sex, older age, and high blood pressure but after multivariate analysis, only male sex was identified as an independent only risk factor. Other authors reported type 2 diabetes [2], older age, and hypertension [19] along with renal insufficiency, preoperative therapeutic anticoagulation, and revisional surgery [20], which was not found in our study.

Most of the patients with bleeding at the JJ had a symptomatology of intestinal obstruction due to an obstructive clot, leading to the distension of the excluded stomach and dilatation of the biliary limb [21]. Left undiagnosed, this complication carries a significant risk of blow-out of the gastric remnant. Abnormal abdominal pain on POD 1 or 2 should raise suspicion of an occlusion of the biliary limb and prompt immediate investigations with CT scan. Once the diagnosis is confirmed, emergency re-laparoscopy is warranted to decompress the excluded stomach with a gastrostomy tube and evacuate blood clots at the JJ using a small enterotomy just distal to it. Failure to first decompress the excluded stomach leads to a risk of massive intraoperative spillage while freeing the intestinal lumen, and carries a significant risk of surgical site infection and sepsis [22, 23]. In patients with ongoing bleeding at the jejunojejunostomy, but without obstruction, selective cautery through a small enterotomy alone is sufficient.

In our series, staplers of 3.5-mm height were used for the stomach and GJ in order to minimize the bleeding events. It has been previously shown that staples height of 3.5 mm compared to 4.8 mm for the circular GJ reduces the risk of anastomotic bleeding [6, 7, 24]. Staplers of 3.5-mm height allow optimal tissue compression, with adequate hemostasis without creating tissue ischemia [6–8, 25]. Oversewing of the staple lines became part of our routine procedure after the initial 70 patients. Staple-line reinforcement using buttressing with glycolic copolymer sleeves and bovine pericardial strips has also been advocated to prevent post-operative bleeding, and some studies concluded to a reduction of bleeding with its use, although none of them was powered enough to allow for definitive conclusions [3, 22, 23]. Although the supremacy of linear over circular stapler for GJ is still discussed, we report the incidence of bleeding which is in the range found in the literature. Moreover, the vast majority of bleeds came from the jejunojejunostomy in our experience, so that we do not have reason to consider circular GJ as inferior with respect to the risk of bleeding. While performing the GJ, we are particularly perceptive about progressive compression of the tissue. We believe that this together with staplers’ height and controlled blood pressure can insure lower risk of bleeding.

We performed JJ anastomosis using a 45-mm-long, 2-row, 2.5-mm staples height until 2010, and with a 45-mm-long, 3-row Tri-Stapler thereafter, with no change in the POB incidence.

Endoscopic control of bleeding at the GJ anastomosis is both diagnostic and a therapeutic option for stable patients with hematemesis and hemoglobin drop. This could be done intraoperatively but takes time, induces additional costs, and has a low yield. Therefore, a careful check before closure of the jejunum is deemed sufficient by most bariatric surgeons. There are three endoscopic treatment options: (i) endoclips, (ii) epinephrine or polidocanol injection, and (iii) thermal methods. Endoclips and epinephrine injections should be favored since thermal methods at the anastomosis induct tissue injury and could increase the likelihood of an anastomotic insufficiency [26].

Although POB was found as the most frequent postoperative complication in this entire series, the present study is limited in its retrospective design and the relatively small incidence of the adverse events studied, which prevents us from drawing definitive conclusions about risk factors. Since preexisting hypertension was found to be a risk factor for bleeding on univariate analysis and is the only modifiable risk factor, we ask our anesthetists to maintain a systolic blood pressure of at least 130 mmHg when performing the anastomoses for optimal hemostasis. During the hospital stay, postoperative hypertension was treated with intravenous beta-blockers or Nitroderm® patch during the first hours after surgery, then rapid resumption of oral medication with crushed tablets as of POD 1. In our experience, vigorous control of the blood pressure during both perioperative and postoperative period is mandatory in order to minimize the risk of anastomotic bleeding.

In conclusion, bleeding after RYGB may occur at different levels of gastrointestinal tract as well as within the abdominal cavity. Successful management is most frequently achieved with conservative measures such as withdrawal of prophylactic anticoagulation, resuscitation and transfusion. Treatment should be tailored depending on suspected location of bleeding and hemodynamic stability. Bleeding at the JJ represents a significant risk of biliary limb obstruction with subsequent dilatation and blowout of the remnant stomach and represents as such a real surgical emergency.

## Abbreviations

*BMI*: Body mass index
*ELB*: Extra-luminal bleeding
*GJ*: Gastrojejunostomy
*ILB*: Intra-luminal bleeding
*ICU*: Intensive care unit
*IPC*: Intermittent pneumatic compression
*JJ*: Jejuno-Jejunal
*LOS*: Length of hospital stay
*OR*: Operating room
*POB*: Postoperative bleeding
*RBC*: Red blood cells
*RYGB*: Laparoscopic Roux-en-Y gastric bypass
*SBO*: Small bowel obstruction
